# Response by sex to statin plus ezetimibe or statin monotherapy: A pooled analysis of 22,231 hyperlipidemic patients

**DOI:** 10.1186/1476-511X-10-146

**Published:** 2011-08-22

**Authors:** Beth L Abramson, Pascale Benlian, Mary E Hanson, Jianxin Lin, Arvind Shah, Andrew M Tershakovec

**Affiliations:** 1 St. Michael's Hospital, Toronto, Ontario, Canada; 2 CHRU-Lille, Université Lille 2, Lille, France; 3 Merck Sharp & Dohme Corp., Whitehouse Station, NJ, USA

**Keywords:** low-density lipoprotein cholesterol, hyperlipidemia, ezetimibe, statin

## Abstract

**Background:**

Despite documented benefits of lipid-lowering treatment in women, a considerable number are undertreated, and fewer achieve treatment targets vs. men.

**Methods:**

Data were combined from 27 double-blind, active or placebo-controlled studies that randomized adult hypercholesterolemic patients to statin or statin+ezetimibe. Consistency of treatment effect among men (n = 11,295) and women (n = 10,499) was assessed and percent of men and women was calculated to evaluate the between-treatment ability to achieve specified treatment levels between sexes.

**Results:**

Baseline lipids and hs-CRP were generally higher in women vs. men. Between-treatment differences were significant for both sexes (all p < 0.001 except apolipoprotein A-I in men = 0.0389). Men treated with ezetimibe+statin experienced significantly greater changes in LDL-C (p = 0.0066), non-HDL-C, total cholesterol, triglycerides, HDL-C, apolipoprotein A-I (all p < 0.0001) and apolipoprotein B (p = 0.0055) compared with women treated with ezetimibe+statin. The odds of achieving LDL-C < 100 mg/dL, apolipoprotein B < 90 mg/dL and the dual target [LDL-C < 100 mg/dL & apoliprotein B < 90 mg/dL] was significantly greater for women vs. men and the odds of achieving hs-CRP < 1 and < 2 mg/L and dual specified levels of [LDL-C < 100 mg/dL and hs-CRP < 2 mg/L] were significantly greater for men vs. women. Women reported significantly more gall-bladder-related, gastrointestinal-related, and allergic reaction or rash-related adverse events (AEs) vs. men (no differences between treatments). Men reported significantly more CK elevations (no differences between treatments) and hepatitis-related AEs vs. women (significantly more with ezetimibe+simvastatin vs. statin).

**Conclusions:**

These results suggest that small sex-related differences may exist in response to lipid-lowering treatment and achievement of specified lipid and hs-CRP levels, which may have implications when managing hypercholesterolemia in women.

## Background

The average lifetime risk for cardiovascular disease (CVD) in women is very high, approaching 1 in 2 [1]. Accordingly, the 2011 update to the Guidelines for Cardiovascular Disease Prevention in Women asserts that nearly all women are at risk for CVD and stresses the importance of CVD prevention and appropriate treatment based on appropriate risk assessment [2]. In addition, the new guidelines lowered the threshold defining high risk to > 10% 10-year risk for CVD. With few exceptions, recommendations for preventive measures for CVD are similar in men and women. For cardiovascular risk reduction, the primary target for men and women is low-density lipoprotein cholesterol (LDL-C) [2,3]. Optimal levels of high-density lipoprotein cholesterol (HDL-C), non-HDL-C, and triglycerides have also been recommended for women [2]. In the general population attainment of recommended lipid levels is suboptimal, and fewer women achieve recommended lipid levels compared with men [4]. Even though women are less likely to be recruited in clinical trials [5,6], there is good evidence showing similar benefits of lipid-lowering treatment in both sexes [2,7]. Despite this, a considerable number of women are undertreated, possibly due to perceived lower risk for CVD in women [2].

Findings from the Women's Health Initiative and the Heart and Estrogen/Progestin Replacement Study underscore the importance of evidence-based practice for CVD prevention in women [8,9]. Elucidation of sex-related tolerability and efficacy of specific lipid-lowering treatments may help provide perspective for evidence-based decision making, tailor preventive interventions based on individual risk and benefit, and increase the number of patients attaining individual treatment goals. The objectives of this analysis were to assess sex-related tolerability and lipid-altering efficacy and achievement of specified lipid and high-sensitivity C-reactive protein (hs-CRP) levels in men and women treated with statin + ezetimibe or statin monotherapy in a broad, pooled database of greater than 21,000 patients.

## Methods

Data were combined from 27 double-blind, active- or placebo-controlled efficacy studies that randomized adult hypercholesterolemic patients to statin alone or statin plus ezetimibe. Studies were conducted from 1999 to 2008 by Merck Research Laboratories to ensure full access to individual patient data (Table 1). Studies with cross-over design, extension studies, studies still ongoing, outcome or imaging studies, studies in which ezetimibe was used as monotherapy or in combination with other non-statin lipid-lowering drugs (e.g., fenofibrate, niacin), adolescent or pediatric patient studies, and studies focusing on patients with sitosterolemia, homozygous familial hypercholesterolemia, aortic stenosis, or chronic kidney disease were not included in the analyses.

**Table 1:** Characteristics of studies included in the pooled analyses

Protocol Number [citation]	Treatment	Randomized to Statin		Randomized to Statin+EZ		Inclusion Criteria	
		
		Men (N = 5389)	Women (N = 5142)	Men (N = 6140)	Women (N = 5607)	Min LDL-C	Max LDL-C
**005** [37]	**PBO, EZ** **EZ+S 10, 20, 40, 80** **S 10, 20, 40, 80**	172	177	169	184	145 mg/dL	250 mg/dL

**011** [38]	**PBO, EZ** **S 10, 20, 40, 80** **EZ+S 10, 20, 40, 80**	110	153	126	148	145 mg/dL	250 mg/dL

**021** [15]	**S 20 + EZ** **Doubling S to 40**	61	49	62	42	101 mg/dL	not specified

**023** [25]	**S 20**, **EZ+S 10, 20, 40**	158	95	292	165	130 mg/dL	not specified

**025** [39]	**A 10** **EZ + S 10, 20**	131	131	279	247	not at LDL-C goal as defined by NCEP ATP III	

**030** [40]	**A 10 + EZ** **Doubling A to 20**	171	145	159	146	130 mg/dL	not specified

**038** [11]	**PBO, EZ** **EZ/S 10, 20, 40, 80** **S 10, 20, 40, 80**	307	315	296	313	145 mg/dL	250 mg/dL

**040** [18]	**Ongoing statin + PBO** **Ongoing statin + EZ**	510	500	1073	947	not at LDL-C goal as defined by NCEP ATP III	

**051** [41]	**EZ/S 10, 20, 40, 80** **A 10, 20, 40, 80**	498	453	496	455	not at LDL-C goal as defined by NCEP ATP III	

**058** [42]	**EZ/S 10, 20, 40, 80** **R 10, 20, 40**	624	857	678	800	145 mg/dL	250 mg/dL

**077** [43]	**EZ/S 20, 40** **A 10, 20, 40**	361	374	221	273	100 mg/dL	not specified

**079** [12]	**A 20 + EZ** **Doubling to A 40**	49	49	58	40	100 mg/dL	160 mg/dL

**090** [16]	**A 40 + EZ** **Doubling to A 80**	178	113	173	115	70 mg/dL	160 mg/dL

**107** [20]	**EZ/S 20, 40** **A 10, 20, 40**	379	307	266	191	70 mg/dL 100 mg/dL	not specified

**112** [22]	**A 10 + EZ** **A 20/40**	249	277	241	286	70 mg/dL 100 mg/dL	160 mg/dL

**679** [44]	**PBO, EZ** **L 10, 20, 40** **EZ+L 10, 20, 40**	88	132	86	106	145 mg/dL	250 mg/dL

**691** [17]	**PBO, EZ** **P 10, 20, 40** **EZ+P 10, 20, 40**	101	104	83	121	145 mg/dL	250 mg/dL

**692** [45]	**PBO, EZ** **A 10, 20, 40, 80** **EZ+A 10, 20, 40, 80**	95	153	107	148	145 mg/dL	250 mg/dL

**700** [46]	**S 20 + EZ** **Double S to 40**	19	15	33	33	130 mg/dL	not specified

**801** [23]	**S 10, 20 + PBO** **S 10, 20 + EZ**	158	52	145	63	101 mg/dL	160 mg/dL

**802** [47]	**S 10, 20 + PBO** **S 10, 20 + EZ**	124	57	124	57	101 mg/dL	160 mg/dL

**803/804** [24]	**A 10, 20 + PBO** **A 10, 20 + EZ**	157	73	153	67	101 mg/dL	160 mg/dL

**806** [48]	**EZ/S 20** **Doubling to A 20**	128	86	141	80	101 mg/dL	160 mg/dL

**807** [49]	**EZ/S 20, 40** **Doubling to A 20**	108	111	224	218	not specified	not specified

**809** [13]	**EZ/S 20** **R10**	185	119	185	129	100 mg/dL	160 mg/dL

**2173/2246** [14]	**Ongoing statin + EZ** **Ongoing statin+ PBO**	221	169	222	157	not at LDL-C goal as defined by NCEP ATP III	

**3377** [21]	**EZ+S 20** **S 20**	47	76	48	76	145 mg/dL	250 mg/dL

A = atorvastatin; EZ = ezetimibe; L = lovastatin; LDL-C = low-density lipoprotein cholesterolemia; NCEP ATP III = National Cholesterol Education Program Adult Treatment Panel III; P = pravastatin = S = simvastatin.

Specific inclusion criteria for the individual studies have been previously published (see citations in Table 1). As guidelines changed over time, there was no single lipid entry criterion that applied to all studies. In general, a patient was considered hypercholesterolemic if LDL-C levels were above guideline-recommended levels according to risk. The range of baseline LDL-C inclusion levels in the studies was > 70 mg/dL to < 250 mg/dL (Table 1). Ezetimibe add-on treatments included ezetimibe 10 mg added to atorvastatin 10-80 mg, ezetimibe 10 mg added to lovastatin 10-40 mg, ezetimibe 10 mg added to pravastatin 10-40 mg, ezetimibe 10 mg added to simvastatin 10-80 mg, and ezetimibe 10 mg added to ongoing statin dose. Statin monotherapy included atorvastatin 10-80 mg, lovastatin 10-40 mg, pravastatin 10-40 mg, rosuvastatin 10-40 mg, and simvastatin 10-80 mg.

Drug-naïve patients were randomized to receive double-blind ezetimibe/statin [ezetimibe/simvastatin combination tablet (10/10, 10/20, 10/40 or 10/80 mg) or ezetimibe 10 mg co-administered with: simvastatin 10, 20, 40 or 80 mg; lovastatin 10, 20 or 40 mg; pravastatin 10, 20 or 40 mg or atorvastatin 10, 20, 40 or 80 mg] or statin alone (simvastatin 10, 20, 40 and 80 mg; lovastatin 10, 20 and 40 mg; pravastatin 10, 20 and 40 mg; atorvastatin 10, 20, 40 and 80 mg or rosuvastatin 10, 20 or 40 mg) for up to 12 weeks. In the add-on therapy studies, statin-treated patients were randomized to receive double-blind placebo or ezetimibe 10 mg administered in combination with their ongoing, previously prescribed, open-label statin (simvastatin 20 or 40 mg or atorvastatin 10, 20, or 40 mg) or doubling the statin dose (to simvastatin 40 or 80 mg or to atorvastatin 20, 40, or 80 mg) for up to 24 weeks. In switch-therapy studies, statin-treated patients were switched from their ongoing, previously prescribed, open-label statin (simvastatin 20 or 40 mg; pravastatin 40 mg; fluvastatin 80 mg; atorvastatin 10 or 20 mg; or rosuvastatin 5 mg) to receive double-blind ezetimibe/statin (ezetimibe/simvastatin combination tablet 10/20 or 10/40 mg) or statin alone (atorvastatin 20 mg or rosuvastatin 10 mg) for up to 6 weeks.

### Efficacy Measures

The % change from baseline to study end in LDL-C, HDL-C, non-HDL-C, total cholesterol, triglycerides, apolipoprotein B, apolipoprotein A-I; ratios for total cholesterol/HDL-C, LDL-C/HDL-C, apolipoprotein B/apolipoprotein A-I, non-HDL-C/HDL-C; and hs-CRP were measured. In addition, the % of patients achieving the single specified targets of LDL-C < 100 mg/dL, non-HDL-C < 130 mg/dL, apolipoprotein B < 90 mg/dL, and specified levels of hs-CRP < 2 mg/L and < 1 mg/L as well as the dual specified levels of LDL-C < 100 mg/dL & non-HDL-C < 130 mg/dL, LDL-C < 100 mg/dL & apolipoprotein B < 90 mg/dL, LDL-C < 100 mg/dL & hs-CRP < 2 mg/L, LDL-C < 100 mg/dL & hs-CRP < 1 mg/L were calculated.

### Laboratory methods

Analysis of samples for all clinical laboratory values were performed at a certified central laboratory according to standards specified by the National Heart Lung and Blood Institute and Centers for Disease Control and Prevention. LDL-C levels were calculated using the Friedewald equation or measured directly using the beta-quantification method if triglycerides were > 400 mg/dL.

### Statistics

Efficacy was assessed in a modified version of the intent-to-treat population, which includes all randomized patients who had baseline values, had taken at least 1 dose of study medication, and had 1 or more post-baseline measurement. Safety was evaluated in all patients as treated; however, laboratory adverse experiences were analyzed in patients who had at least 1 post-baseline assessment.

Consistency of treatment effect among men and women was tested with analysis of covariance with terms for treatment, first-/second-line lipid-lowering therapy status, race, sex, coronary heart disease (CHD), statin potency, age, body mass index, baseline LDL-C, baseline HDL-C, baseline triglycerides, baseline response variable, diabetes, trial within first-/second-line lipid-lowering therapy status, treatment by first-/second-line lipid-lowering therapy status, and treatment by sex interactions. Statistical tests were two-sided and a p-value of less than 0.05 was considered significant. However, due to the large population size, even very small differences may be statistically significant, although not clinically relevant. Therefore, inferential statistics were not included for baseline values.

Statin potency was defined on the basis of LDL-C reduction as low potency: simvastatin 10 mg, lovastatin 10-20 mg, pravastatin 10-20 mg, fluvastatin 10-40 mg, and cerivastatin 0.2-0.3 mg; medium potency: simvastatin 20-40 mg, atorvastatin 10-20 mg, lovastatin 40-80 mg, pravastatin 40-80 mg, fluvastatin 80-160 mg, cerivastatin 0.4-0.8 mg, and rosuvastatin 5 mg; high potency: simvastatin 80 mg, atorvastatin 40-80 mg, rosuvastatin 10-40 mg. The % of men and women, adjusted odds ratios, and 95% confidence intervals (CIs) were calculated using a logistic model with terms for first-/second-line therapy, gender, treatment, and baseline value to evaluate the between-treatment ability to achieve specified treatment levels in the full cohort and with terms for first-/second-line therapy, treatment, and baseline value in each sex subgroup. No adjustments were made for multiplicity. Due to the large population size, the results of these analyses should be interpreted with caution, since statistical significance may not always imply clinical significance.

## Results

A total of 22,231 patients were included in the safety analysis and 21,794 patients were included in the modified intent-to-treat population for the efficacy analysis. Subjects received the following active treatment regimens during the studies: simvastatin: n = 2148; atorvastatin: n = 4433; rosuvastatin: n = 1725; lovastatin: n = 220; pravastatin: n = 205; unspecified statin: n = 1577; ezetimibe+simvastatin: n = 2372; ezetimibe+atorvastatin: n = 4226; ezetimibe+rosuvastatin: n = 1723; ezetimibe+lovastatin: n = 192; ezetimibe+pravastatin: n = 205; ezetimibe+unspecified statin: n = 2760 (Table 1).

Baseline demographics were generally similar, although risk factors differed somewhat between the sexes: More women vs. men had body mass index ≥30 kg/mg^2^, more women vs. men had metabolic syndrome or diabetes mellitus, more men vs. women had CHD, and at study entry, more men vs. women were being treated with ongoing statin therapy (Table 2). Baseline lipid and hs-CRP levels were generally higher in women compared with men, except for lipid ratios, which were generally similar between sexes (Table 2).

**Table 2:** Baseline demographics, risk factors and clinical characteristics

	Male		Female	
	**Statin** **(n = 5279)**	**Statin + EZ** **(n = 6016)**	**Statin** **(n = 5029)**	**Statin + EZ** **(n = 5470)**

Mean age, years (SD)	58.4 (11.22)	59.2 (11.14)	60.0 (11.23)	60.7 (11.01)
Age, n (%)				
< 65 years	3556 (67.4)	3909 (65.0)	3126 (62.2)	3341 (61.1)
65-74 years	1361 (25.8)	1614 (26.8)	1457 (29.0)	1557 (28.5)
≥75 years	362 (6.9)	493 (8.2)	446 (8.9)	572 (10.5)
Caucasian, n (%)	4549 (86.2)	5187 (86.2)	4141 (82.3)	4519 (82.6)
BMI < 30 kg/m^2^ (%)	3287 (63.2)	3728 (62.8)	2721 (55.0)	3056 (56.7)
CHD, n (%)	2129 (40.3)	2614 (43.5)	1181 (23.5)	1501 (27.5)
Diabetes, n (%)	1491 (28.2)	1732 (28.8)	1591 (31.6)	1727 (31.6)
Metabolic syndrome	1912 (46.5)	2112 (45.8)	2321 (54.7)	2406 (53.6)
Ongoing Statin*, n (%)	2271 (43.0)	2935 (48.8)	1780 (35.4)	2310 (42.2)

Least squares mean (SD)	**(n = 5279)**	**(n = 6016)**	**(n = 5029)**	**(n = 5470)**

LDL-C (mg/dL)	147.0 (40.3)	144.4 (39.8)	156.4 (41.7)	154.2 (42.5)
HDL-C (mg/dL)	45.8 (10.5)	45.7 (10.3)	53.4 (12.7)	53.7 (13.0)
non-HDL-C (mg/dL)	180.2 (44.5)	177.5 (44.0)	190.1 (45.8)	187.6 (46.4)
TC (mg/dL)	226.0 (44.0)	223.2 (43.6)	243.6 (45.7)	241.3 (46.2)
Triglycerides (mg/dL) ^‡^	150.0 (88.1)	151.0 (87.9)	156.5 (90.2)	154.0 (90.7)
Apo B (mg/dL)	142.5 (32.7)	141.3 (32.3)	147.9 (34.0)	146.4 (34.4)
Apo AI (mg/dL)	146.3 (23.2)	146.4 (23.8)	164.2 (27.5)	165.0 (28.0)
hs-CRP (mg/L) ^‡^	1.8 (2.7)	1.8 (2.7)	2.7 (4.1)	2.8 (4.1)
non-HDL-C/HDL-C	4.1 (1.5)	4.1 (1.4)	3.8 (1.4)	3.7 (1.4)
Apo B/Apo AI	1.0 (0.3)	1.0 (0.3)	0.9 (0.3)	0.9 (0.3)
LDL-C/HDL-C	3.4 (1.2)	3.3 (1.2)	3.1 (1.1)	3.0 (1.1)
TC/HDL-C	5.2 (1.5)	5.1 (1.4)	4.8 (1.4)	4.7 (1.4)

Apo = Apolipoprotein; BMI = body mass index; CHD = coronary heart disease; EZ = ezetimibe; LDL-C = low-density lipoprotein cholesterol; HDL-C = high-density lipoprotein cholesterol; hs-CRP = high-sensitivity C-reactive protein; SD = standard deviation; TC = total cholesterol

*Patients who entered a study who were already receiving statin treatment prior to screening

^‡^presented as median values (robust SD); p-values based on ANOVA model using Tukey scores transformed values with term for gender.

**Figure 1:**
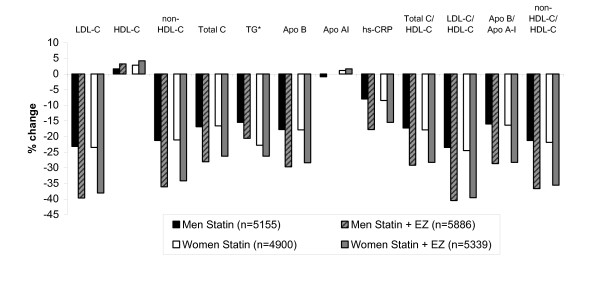
**Percent change in lipid, lipid ratio, and hs-CRP levels in male and female patients**. All between-treatment differences were p < 0.0001 except for Apo A-I, for which the between treatment difference in women was p = 0.0009 and in men was p = 0.0389.

As seen in the individual studies, the overall population demonstrated significant reductions in LDL-C, non-HDL-C, triglycerides, apolipoprotein B, and hs-CRP. Significantly greater reductions were observed in subjects treated with combination statin + ezetimibe vs. statin monotherapy (all p < 0.0001; data not shown). All between-treatment differences were statistically significant for both sexes (all p < 0.001 except apolipoprotein A-I in men = 0.0389; Figure 1). Lipid responses to statins were similar for both sexes; however, men who received ezetimibe + statin combination experienced a significantly greater change in LDL-C (p = 0.0066), non-HDL-C, total cholesterol, triglycerides, HDL-C, apolipoprotein A-I (all p < 0.0001) and apolipoprotein B (p = 0.0055) compared with women who received ezetimibe + statin combination (Figure 1), although these differences were small (≤2%) and the clinical relevance could be debated. There was no significant effect of sex on the changes in the lipid ratios or hs-CRP (all p > 0.05; Figure 1).

**Figure 2:**
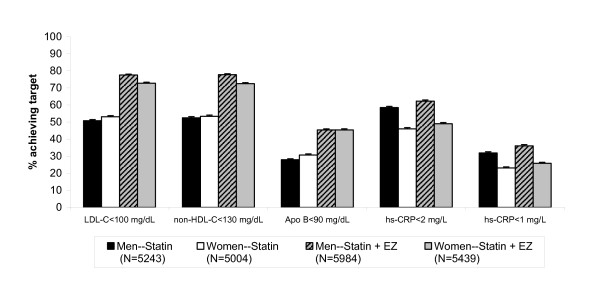
**Percent of patients by sex achieving specified single lipid and hs-CRP levels**. Error bars represent standard error.

**Figure 3:**
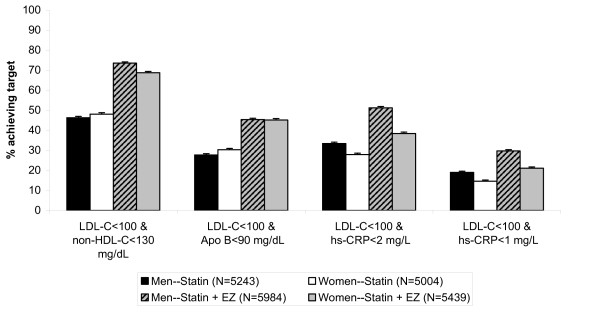
**Percent of patients by sex achieving dual specified lipid and hs-CRP levels**. Error bars represent standard error.

Figures 2 and 3 show the percentage of patients achieving single and dual lipid and hs-CRP levels by sex. When comparing men vs. women, the odds of achieving LDL-C < 100 mg/dL, apolipoprotein B < 90 mg/dL and the dual target [LDL-C < 100 mg/dL & apolipoprotein B < 90 mg/dL] was significantly greater for women, although the differences were small (Table 3). Conversely, the odds of achieving hs-CRP < 1 mg/L, hs-CRP < 2 mg/L and dual specified levels of [LDL-C < 100 mg/dL and hs-CRP < 2 mg/L] were significantly greater for men than for women, and those differences were also small (Table 3). Of note, the odds ratios and 95% confidence intervals (Table 3) are calculated from the model with terms for first-/second-line therapy, sex, treatment and baseline LDL-C. The baseline is adjusted and the statistics are comparing all women versus all men attaining specified targets, and not by treatment group. When comparing by treatment group in both men and women, the odds of achieving all single and dual specified targets was greater in subjects treated with the combination statin + ezetimibe compared with statin monotherapy (Table 4).

**Table 3:** Adjusted odds ratios and 95% confidence intervals* in females vs males

	Female vs. Male
LDL-C < 100 mg/dL	**1.09 (1.02, 1.17)**
non-HDL-C < 130 mg/dL	1.03 (0.96, 1.10)
Apo B < 90 mg/dL	**1.14 (1.07, 1.22)**
hs-CRP < 2 mg/L	**0.76 (0.71, 0.82)**
hs-CRP < 1 mg/dL	**0.86 (0.80, 0.93)**
LDL-C < 100 & non-HDL-C < 130 mg/dL	1.07 (1.00, 1.14)
LDL-C < 100 & Apo B < 90 mg/dL	**1.11 (1.04, 1.19)**
LDL-C < 100 & hs-CRP < 2 mg/L	**0.90 (0.84, 0.97)**
LDL-C < 100 & hs-CRP < 1 mg/L	0.99 (0.91, 1.07)

Apo = Apolipoprotein; EZ = ezetimibe; LDL-C = low-density lipoprotein cholesterol; HDL-C = high-density lipoprotein cholesterol; hs-CRP = high-sensitivity C-reactive protein

*Ratio of the predictive odds of attaining target in female versus male patients is based on the logistic model with terms for first/second line, sex, treatment, and baseline value(s).

**Table 4:** Adjusted odds ratios and 95% confidence intervals for between-treatment comparisons*

LDL-C < 100 mg/dL	5.29 (4.79, 5.84)
non-HDL-C < 130 mg/dL	5.08 (4.59, 5.61)
Apo B < 90 mg/dL	3.30 (2.98, 3.64)
hs-CRP < 2 mg/L	1.22 (1.12, 1.34)
hs-CRP < 1 mg/dL	1.26 (1.15, 1.39)
LDL-C < 100 & non-HDL-C < 130 mg/dL	5.18 (4.70, 5.70)
LDL-C < 100 & Apo B < 90 mg/dL	3.34 (3.02, 3.69)
LDL-C < 100 & hs-CRP < 2 mg/L	2.52 (2.30, 2.77)
LDL-C < 100 & hs-CRP < 1 mg/L	2.05 (1.84, 2.28)

Apo = Apolipoprotein; EZ = ezetimibe; LDL-C = low-density lipoprotein cholesterol; HDL-C = high-density lipoprotein cholesterol; hs-CRP = high-sensitivity C-reactive protein

*Ratio of the predictive odds of attaining target on Statin+EZ versus statin based on the logistic model with terms for first/second line, treatment, and baseline value(s).

A summary of adverse events is listed in Table 5. When comparing the full cohort, both treatments had generally similar tolerability and safety profiles (i.e., there were no between-treatment differences in adverse events, liver enzymes, or creatine kinase elevations). There were significantly more alanine aminotransferase (ALT) elevations ≥3 × upper limit of normal reported in the combination treatment group compared with statin monotherapy, and although there was no statistically significant effect of sex, this difference was primarily driven by elevations in males (Table 5). When comparing the sexes, women reported significantly more adverse events (i.e., ≥1 adverse event), drug-related adverse events, gall-bladder-related adverse events, gastrointestinal-related adverse events, allergic reaction or rash-related adverse events; and more women discontinued due to adverse events and drug-related adverse events, although there were no significant differences between treatments (Table 5). Compared with women, men reported significantly more creatine kinase elevations ≥ 10 × upper limit of normal (no differences between treatments) and hepatitis-related adverse events, of which there were significantly more in the combination group vs. the statin monotherapy group (Table 5). There were 7 cases of myopathy reported, with no significant differences between treatments or sexes in their occurrence, and no cases of rhabdomyolysis reported (Table 5). A total of 12 deaths were reported (5 in the statin group and 7 in the statin + ezetimibe group) during the course of all 27 studies and none of the deaths were attributed to treatment.

**Table 5:** Adverse event summary

	Full Cohort		Male				Female			
**Adverse events, n (%)**	**Statin**	**Statin/EZ**	**Between treatment p-value**	**P-value for effect of sex**	**Statin**	**statin/EZ**	**Between** **Treatment** **p-value**	**Statin**	**Statin/EZ**	**Between** **treatment** **p-value**
	**N = 10517**	**N = 11714**			**N = 5380**	**N = 6129**		**N = 5137**	**N = 5585**	

≥1	3455 (32.9)	3717 (31.7)	0.849	< 0.001	1537 (28.6)	1779 (29.0)	0.173	1918 (37.3)	1938 (34.7)	0.114
Drug related*	833 (7.9)	961 (8.2)	0.181	< 0.001	349 (6.5)	456 (7.4)	0.025	484 (9.4)	505 (9.0)	0.819
Serious	145 (1.4)	187 (1.6)	0.220	0.370	74 (1.4)	107 (1.7)	0.122	71 (1.4)	80 (1.4)	0.091
Serious drug related	6 (0.1)	13 (0.1)	0.148	0.908	2 (0.0)	8 (0.1)	0.075	4 (0.1)	5 (0.1)	0.784
Discontinuations^† ^due to AEs	219 (2.1)	263 (2.2)	0.286	< 0.001	91 (1.7)	105 (1.7)	0.828	128 (2.5)	158 (2.8)	0.229
Drug related*	136 (1.3)	177 (1.5)	0.117	< 0.001	49 (0.9)	69 (1.1)	0.234	87 (1.7)	108 (1.9)	0.288
Serious	34 (0.3)	38 (0.3)	0.906	0.320	18 (0.3)	23 (0.4)	0.659	16 (0.3)	15 (0.3)	0.746
Serious drug related	6 (0.1)	7 (0.1)	0.864	0.749	2 (0.0)	4 (0.1)	0.485	4 (0.1)	3 (0.1)	0.689
Deaths	5 (0.0)	7 (0.1)	0.702	0.655	4 (0.1)	3 (0.0)	0.582	1 (0.0)	4 (0.1)	0.197
Gastrointestinal-related^‡^	861 (8.2)	889 (7.6)	0.367	< 0.001	339 (6.3)	390 (6.4)	0.636	522 (10.2)	499 (8.9)	0.113
Gallbladder-related^§^	9 (0.1)	11 (0.1)	0.824	0.014	2 (0.0)	3 (0.0)	0.754	7 (0.1)	8 (0.1)	0.940
Allergic reaction or rash^║^	175 (1.7)	213 (1.8)	0.194	< 0.001	70 (1.3)	90 (1.5)	0.326	105 (2.0)	123 (2.2)	0.382
Hepatitis-related^¶^	24 (0.2)	39 (0.3)	0.079	0.030	13 (0.2)	27 (0.4)	0.038	11 (0.2)	12 (0.2)	0.858
ALT ≥ 3 × ULN, consecutive, m/n (%)	31/10341 (0.3)	50/11512 (0.4)	0.519	0.241	15/5289 (0.3)	34/6031 (0.6)	0.111	16/5052 (0.3)	16/5481 (0.3)	0.611
AST ≥ 3 × ULN, consecutive, m/n (%)	23/10342 (0.2)	30/11512 (0.3)	0.087	0.100	7/5290 (0.1)	16/6031 (0.3)	0.018	16/5052 (0.3)	14/5481 (0.3)	0.873
ALT or AST ≥3 × ULN, consecutive, m/n (%)	36/10342 (0.3)	64/11512 (0.6)	0.018	0.185	17/5290 (0.3)	41/6031 (0.7)	0.006	19/5052 (0.4)	23/5481 (0.4)	0.649
CK ≥ 10 × ULN, m/n (%)	13/10342 (0.1)	9/11514 (0.1)	0.337	0.028	9/5290 (0.2)	7/6033 (0.1)	0.543	4/5052 (0.1)	2/5481 (0.0)	0.395
Myopathy^#^, m/n (%)	4/10342 (0.04)	3/11512 (0.03)	0.667	0.699	2/5290 (0.04)	2/6031 (0.02)	0.985	2/5052 (0.04)	1/5481 (0.02)	0.517
Rhabdomyolysis^, m/n (%)	0/10342 (0.00)	0/11512 (0.00)	N/A	N/A	0/5290 (0.00)	0/6031 (0.00)	N/A	0/5052 (0.00)	0/5481 (0.00)	N/A

ALT = alanine aminotransferase, ALT = aspartate aminotransferase, CK = creatine kinase ULN = upper limit of normal

% = m/n × 100 = (number of patients within the AE category/number of treated patients) × 100

*Determined by the investigator to be related to the drug

^†^Study medication withdrawn

^‡^For gastrointestinal-related clinical adverse events, the preferred terms (preferred Medra terms) were pre-identified for collective review, including e.g. abdominal discomfort, abdominal distension, abdominal pain, abdominal tenderness, colitis, colonic polyp, constipation, dental caries, dental discomfort, diarrhoea, diverticulum, duodenitis, dyspepsiea, dysphagia, erosive duodenitis, faeces discolored, flatulence, flood poisoning, gastitis, gastoesophageal reflux disease, gingival pain, haemorrhoids, hiatus hernia, nausea, oesophageal stenosis, rectal haemorrhage, tooth loss, toothache, and vomiting.

^§^For gallbladder-related clinical adverse events the preferred terms (preferred Medra terms) were pre-identified for collective review, including e.g. bile duct obstruction, bile duct stenosis, bile duct stone, biliary colic, cholangitis, cholecystectomy, cholecystitis, cholelithiasis, gallbladder disorder, and gallbladder perforation

^║^For allergic reaction or rash the preferred terms (preferred Medra terms) were pre-identified for collective review, including e.g. analphylaxis, angioedema, dermatitis, dermagraphism, drug hypersensitivity, eczema, eosinophilia, erythema, face oedema, hypersensitivity, palmar erythema, periorbital oedema, photodermatosis, photosensitivity, pigmentation disorder, pruritis, rash, rosacea, skin disorder, skin exfoliation, skin hyperpigmentation, skin inflammation, skin lesion, subcutaneious nodule, systemic lups erythematosus rash, and urticaria

^¶^In addition to review of the effects of ezetimibe + statin on laboratory parameters associated with liver function, potentially "hepatitis-related" clinical adverse event terms (preferred Medra terms) were pre-identified for collective review, e.g., cholestasis, hepatic necrosis, hepatocellular injury, cytolytic hepatitis, hepatitis, hepatomegaly, hepatic cyst, hepatitis cholestatic, jaundice, hepatic failure, hepatitis fulminant, jaundice cholestatic, hepatic lesion, and, hepatitis infectious.

^#^Myopathy is defined as CK elevation > 10XULN with associated muscle symptoms with no other explanatory cause.

^Rhabdomyolysis is defined as myopathy with associated evidence of renal damage.

## Discussion

The results of this pooled analysis demonstrated that in general, the response to statins was similar for both men and women. Men had slightly greater lipid responses to the ezetimibe + statin combination compared with women, but the clinical relevance of this is questionable. When comparing women and men, the odds were greater for women vs. men in the attainment of specified LDL-C and apolipoprotein B targets, while the odds of achieving specified hs-CRP targets was greater for men vs. women. In the full cohort and in both sexes, achievement of specified single and dual lipid levels was greater with statin + ezetimibe vs. statin. The tolerability profiles were generally similar between sexes and treatments. The differences reported here are small. In addition, there was no significant effect of sex on the changes in the lipid ratios, supporting the perspective that the small differences in LDL-C response observed are not likely to translate into clinically meaningful differences. These findings suggest any potential sex differences that may exist in response to lipid-lowering therapy and achievement of specified lipid and hs-CRP levels, should unlikely account for the care gaps reported in the literature [4,10]. These data should be considered when planning and assessing such treatment in men and women.

Analyses of changes in LDL-C by sex subgroups have been presented in several previous reports and demonstrated that men and women responded to treatment similarly to the overall population [11-22]. In those studies, the subgroups by sex were relatively small; and although the analyses of change in LDL-C were prespecified, they were not powered to show statistical differences between the sex subgroups. Moreover, previous reports of individual studies have demonstrated no significant treatment by sex interactions for LDL-C target attainment [23-25]. The results in the current pooled analysis showed that women are ~9% more likely to achieve LDL-C < 100 mg/dL than men if they were in the same first-line or second-line study with the same treatment and similar baseline LDL-C. It is important to note that this analysis was a comparison of all women vs. all men attaining LDL-C targets, and was not a between-treatment comparison. This result may seem unusual in light of the slightly greater LDL-C reductions observed in men vs. women with ezetimibe + statin and higher baseline LDL-C levels observed in women vs. men. However, the model accounted for baseline factors, including LDL-C level, first-line or second-line study, and treatment. Since the model accounts for the other factors, such as study type, the presence of diabetes or CHD, and baseline LDL-C, further study may elucidate the factors associated with greater achievement of lipid targets.

Also of interest in the current analysis were the differences in the odds of achieving specified lipid and hs-CRP levels between the sexes. There were small but significant differences between men and women in the odds of attaining single specified apolipoprotein B and hs-CRP levels; the most notable of these differences being the greater odds for men vs. women achieving both specified levels of hs-CRP. The magnitude of difference between treatment groups for attainment of specified hs-CRP levels was relatively small compared with other lipid levels, and there were clear differences between the sexes in achievement of both specified hs-CRP levels. Higher baseline levels of hs-CRP in women could also play a role in lower achievement in women vs. men. One could speculate that the higher levels of hs-CRP in women, both at baseline and study end, could be due to the higher prevalence of metabolic syndrome and obesity in women, the presence of which has been associated with increased levels of hs-CRP and other inflammatory markers [26].

Lipid-lowering treatment reduces coronary events [27], although the consistency of this effect in women has been controversial until recently [28,29]. Stronger contemporary data have provided some evidence toward answers to that debate. A meta-analysis of primary prevention trials that included sex-specific clinical outcomes in CVD demonstrated that cardiovascular events were reduced by about one-third in women after 12 months of statin treatment, during which subjects experienced a 51 mg/dL reduction from baseline in LDL-C [7]. Similar relative risk reductions were observed in men. The Cholesterol Treatment Trialists' (CTT) Collaboration showed that further reductions in LDL cholesterol produce definite further reductions in the incidence of cardiovascular events in the overall population, with a significant proportional risk reduction of 17% (p < 0.001) per 39 mg/dL reduction in LDL-C among women in first major vascular events [30]. Taken together, these data support the utility of intensive lipid lowering for reducing the risk of cardiovascular events in both men and women. Though the ultimate assessment of clinical impact of lipid-lowering therapy supported by ezetimibe-related therapy awaits results of IMPROVE-IT, [31] results from SEAS and SHARP describe lower rates of ischemic cardiac events with ezetimibe-simvastatin therapy in subjects with aortic stenosis and chronic kidney disease, respectively [32-34].

Both treatments were generally well tolerated in the overall population and in both sexes. This is consistent with previous reports, which have shown generally comparable safety and tolerability profiles with statin monotherapy and ezetimibe + statin coadministration treatment [35,36]. In conclusion, the results of the present study underscore the utility of lipid-lowering therapy in women. The small sex-related differences in lipid-lowering therapy may not be clinically meaningful and underscore the ongoing need for appropriate management of lipid levels in women.

## Abbreviations

ALT: alanine aminotransferase; CHD: coronary heart disease; CIs: confidence intervals: CVD: cardiovascular disease; HDL-C: high-density lipoprotein cholesterol; hs-CRP: high-sensitivity C-reactive protein; LDL-C: low-density lipoprotein cholesterol.

## Competing interests

BLA has received speaker fees for various CMEs sponsored by pharmaceutical companies including Merck Sharp & Dohme Corp.

PB has received payment as a consultant and for lectures on behalf of Merck/Schering-Plough; and payment for development of educational presentations for Astra Zeneca.

MEH, JL, AS, and AMT are employees of Merck Sharp & Dohme Corp and may own stock or hold stock options in the company.

## Authors' contributions

BLA performed or supervised analyses and interpreted results, provided substantive suggestions for revision, or critically reviewed the manuscript. PB conceived, designed or planned the study, interpreted the results, and provided substantive suggestions for revision or critically reviewed the manuscript. MEH conceived, designed or planned the study, interpreted the results, wrote sections of the initial draft, and provided substantive suggestions for revision or critically reviewed the manuscript. JL collected or assembled the data, performed or supervised analyses and interpreted the results; provided substantive suggestions for revisions or critically reviewed the manuscript; provided statistical expertise. AS performed or supervised analyses and interpreted the results; provided substantive suggestions for revisions or critically reviewed the manuscript; provided statistical expertise. AMT conceived, designed or planned the study and interpreted the results; and provided substantive suggestions for revisions or critically reviewed the manuscript. All authors reviewed and approved the final version of the paper.
